# Mathematical Modeling of Obstetric Variables: Influence of COVID-19, Periodontal Disease and Dental Care During Pregnancy

**DOI:** 10.3390/biomedicines13122919

**Published:** 2025-11-28

**Authors:** Juliana Velosa-Porras, Sandra Catalina Correa Herrera, Katherine Lucia Mejía Reyes, Paula Sofía Fuentes Rojas, Laura Daniela Ardila Ortiz, Olga Lucía Ospina, Signed Prieto-Bohórquez, Jairo Javier Jattin Balcázar, Jorge Edgar Guevara Muñoz, Leonardo Bonilla Cortés, Javier M. Mora-Méndez, Catalina Latorre Uriza, Francina María Escobar Arregoces, Nelly S. Roa

**Affiliations:** 1 Centro de Investigaciones Odontológicas, Faculty of Dentistry, Pontificia Universidad Javeriana, Bogotá 110231, Colombia; clatorre@javeriana.edu.co (C.L.U.); escobar.f@javeriana.edu.co (F.M.E.A.); 2 Grupo de Investigación Armonía, Centro de Investigación y Atención Psicosocial Hanami, Bogotá 110231, Colombia; scatalinacorreah@gmail.com (S.C.C.H.); danielaardila204@gmail.com (L.D.A.O.); u0401578@unimilitar.edu.co (J.J.J.B.); 3 Graduate Program, Department of Periodontology, Faculty of Dentistry, Pontificia Universidad Javeriana, Bogotá 110231, Colombia; katherinemejiar@javeriana.edu.co (K.L.M.R.); paulasofiafuro@gmail.com (P.S.F.R.); 4 Grupo de Biofísica y Bioquímica Estructural, Departamento de Física, Faculty of Science, Pontificia Universidad Javeriana, Bogotá 110231, Colombia; olucia@javeriana.edu.co; 5 Independent Researcher, Bogotá 110231, Colombia; insightgroup.ext@gmail.com; 6 Compensar Salud EPS, Bogotá 110231, Colombia; jeguevaram@compensarsalud.com; 7 Unidad de Obstetricia y Ginecología, Hospital Universitario Clínica San Rafael, Bogotá 110231, Colombia; docleobonilla@yahoo.com; 8 Departamento de Docencia e Investigación, Hospital Universitario Clínica San Rafael, Bogotá 110231, Colombia; javier.mora@stewardcolombia.org

**Keywords:** periodontitis, COVID-19, pregnancy, dental care, obstetric outcomes, oral health, set theory, probability

## Abstract

**Background:** Systemic inflammatory factors may be altered by periodontitis and/or COVID-19, potentially increasing the risk of adverse pregnancy outcomes, a relationship that remains unclear. **Objective:** This study aimed to identify associations between periodontitis and COVID-19 during pregnancy, evaluating the influence of dental care on obstetric variables through set theory and probability. **Methods:** A quantitative, cross-sectional, and correlational study was conducted in two phases. The first phase analyzed 156 medical records from 5 institutions, including gynecological and periodontal data; the second phase examined 104 records from a single institution selected for data completeness (2020–2021). Descriptive statistics, bivariate analysis, chi-square tests, and odds ratios were applied. Set operations (union, intersection) and relative probabilities were calculated using R and Excel. Sets represented dental care, dental disease, COVID-19 diagnosis, gestational age, neonatal weight, and complications. **Results:** In Phase 1, 37% of pregnant women were COVID-19-positive, 44% vaccinated, 51.9% underwent cesarean section, and 5.12% had periodontitis. In Phase 2, 76 pregnant women did not receive dental care, while 28 did; among them, 6 were COVID-19-positive. Mean neonatal weight ranged from 2336 g (dental care) to 2271 g (no dental care). COVID-19-positive pregnant women showed fewer complications and a higher proportion of normal-weight neonates. Gingivitis was the most frequent periodontal condition (75%). No statistically significant differences were observed between the analyzed sets. Conclusions: no direct relationship was found between periodontitis and neonatal weight in COVID-19-positive cases. Dental care did not influence maternal–fetal outcomes. The methodology provides an innovative framework for clinical analysis through mathematical abstraction.

## 1. Introduction

Preterm birth, defined as delivery occurring between 22 and 36 weeks of gestation, remains a major global public health concern. Its prevalence is estimated at approximately 11% with an incidence of 9.6%, representing nearly 12.9 million preterm infants annually. This condition is the second leading cause of mortality in children under five years of age, accounting for over one million deaths per year due to complications related to prematurity. Moreover, preterm birth has been associated with long-term consequences such as growth impairments, hearing deficits, and learning development disorders [1,2,3,4,5].

During pregnancy, the increase in cytokines stimulates prostaglandin synthesis in gestational tissues, thereby contributing to the induction of labor by promoting increased uterine contractions and activating inflammatory pathways that ultimately lead to childbirth and the delivery of the newborn [6,7,8,9]. Several studies have demonstrated that conditions within the feto-placental unit may be associated with bacterial colonization of the placenta, amniotic fluid, membranes, fetal circulation, and tissues by periodontal pathogens. This microbial invasion can modulate both local and systemic inflammatory response, potentially triggering adverse outcomes such as preterm birth or miscarriage [10,11,12,13,14,15,16,17,18]. Recently, Latorre et al. reported that periodontal disease can induce a systemic inflammatory response in pregnant women, characterized by elevated levels of prostaglandins such as PGE2 and proinflammatory cytokines [2]. Observational studies and recent systematic reviews support this association between periodontitis in pregnant women and an increased risk of preterm birth and low birth weight [19,20,21,22,23].

During the COVID-19 pandemic, the likelihood of preterm birth among pregnant women infected with SARS-CoV-2 was 21.96% (95% CI: 18.91–25.38), suggesting a considerable increase compared to the estimated proportion in the general population, although it could not be established as a direct consequence of the infection [24,25]. Nevertheless, some studies did not confirm this association [26]. In severe cases of COVID-19, a “cytokine storm” was documented [27], characterized by elevated plasma levels of IL-2, IL-6, IL-7, IL-10, granulocyte colony-stimulating factor (G-CSF), IFN-γ, inducible protein 10 (IP-10/CXCL10), monocyte chemoattractant protein 1 (MCP-1), macrophage inflammatory protein 1 alpha (MIP-1α), and TNF-α, reflecting an exacerbated immune activation with potential implications for the normal progression of pregnancy. Accordingly, one study found that pregnant women with COVID-19 had a higher risk of preterm birth, admission to intensive care units, and the need for mechanical ventilation [28].

Notably, SARS-CoV-2 can induce a “cytokine storm”, an exaggerated immune response that may exacerbate the normal inflammatory state of pregnancy and contribute to obstetric complications by intensifying systemic inflammation [29]. Moreover, elevated levels of TNF-α in maternal peripheral blood have been associated with embryotoxicity and may induce preterm labor in animal models, including primates [29].

This upregulation may increase the susceptibility of pregnant women to SARS-CoV-2 infection. Such vulnerability has been associated with complications including preterm birth and premature rupture of membranes [24,29,30,31]. From this perspective, it has been proposed that the concurrent presence of COVID-19 and periodontal disease in pregnant women may represent an additional risk factor for preterm birth [32,33]; however, this association has not yet been fully investigated or confirmed.

Currently, various studies have explored the relationship between periodontal disease and COVID-19 [34,35], COVID-19 and maternal and neonatal outcomes [36,37,38], and periodontal disease and maternal and neonatal outcomes [39,40]. However, to date, no study has examined the intersection of all three conditions: periodontal disease, COVID-19, and pregnancy. Furthermore, despite some controversy among previous research findings, dental care was considered in this study, as evidence from observational studies suggests that maintaining adequate oral health during pregnancy may reduce the risk of adverse pregnancy outcomes [39,41]. This study was conducted during the COVID-19 pandemic, a period during which dental clinics remained closed due to globally imposed restrictions and the high risk of cross-contamination with SARS-CoV-2.

The purpose of this study was to identify the relationship between periodontal disease and COVID-19 infection during pregnancy and to analyze how oral conditions, such as periodontal disease, influence clinical variables such as gestational age at delivery, neonatal birth weight, and complications during pregnancy and delivery. Based on the previous objective, we posed the following research question: What is the relationship between COVID-19 during pregnancy, periodontal disease, and dental care in terms of clinical variables such as gestational age at delivery, neonatal birth weight, and complications during pregnancy and delivery?

## 2. Materials and Methods

### 2.1. Study Design and Initial Considerations

A study was conducted using a quantitative approach, with a non-experimental cross-sectional design and a correlational scope. In this design, data were collected at a single point in time to analyze the relationship between the variables without manipulating them. Based on retrospective medical and dental institutional records of pregnant women from the 2020–2021 period, the following characteristics were analyzed: periodontal assessment—periodontal disease, COVID-19 diagnosis, dental care, gestational age at birth, neonatal birth weight, pregnancy and delivery complications, and other dental conditions.

### 2.2. Ethical Considerations

The research protocol was submitted to and approved by the Ethics Committee of the Hospital Universitario Clínica San Rafael (HUCSR) and the Research and Ethics Committee of the Faculty of Dentistry of the Pontificia Universidad Javeriana (CIEFOPUJ) under minutes CEI-185-2021 and OD-0301, respectively.

### 2.3. Population and Sample

Non-probabilistic convenience sampling was employed due to the specific conditions of the research context. Pregnant women over 16 years of age were included if they had received dental care at any point during pregnancy, presented respiratory symptoms, and attended emergency services where a diagnostic test for COVID-19 was performed.

Initially, an exploratory study was conducted to identify which of the participating clinics and hospitals had the highest number of pregnant women with complete gynecological and dental institutional records. This was particularly relevant due to the restrictions imposed during the COVID-19 pandemic, which limited dental services exclusively to emergency care.

The first part (Stage 1) study population consisted of all pregnant women who attended and were treated by EPS Compensar in hospitals located in Bogotá-Colombia, specifically at Hospital Universitario Clínica San Rafael (HUCSR), Los Cobos Medical Center S.A.S., Hospital Universitario San Ignacio, and Clínica La Magdalena during the 2020–2021 period (*n* = 156). All previously described characteristics were analyzed, along with additional demographic variables such as participant age, socioeconomic status, place of residence, vaccination status, type of vaccine received, and systemic health conditions (preeclampsia, hypertension, hypothyroidism, obesity, hypotension, diabetes, insulin resistance, Hodgkin lymphoma, retinal left thrombosis, and Sjögren syndrome).

The second part (Stage 2) of the study focused on medical records from HUCSR that documented dental care, including both general dentistry and periodontics (*n* = 104). This stage was divided into two phases.

In the first phase, the groups of pregnant women who received dental care and those who did not were described and compared, considering neonatal variables such as birth weight and gestational age at delivery.

In the second phase of the study, which exclusively targeted pregnant women who received dental care during gestation, clinical and obstetric outcomes were systematically assessed. The maternal and neonatal clinical variables analyzed included neonatal birth weight, gestational age at delivery, maternal COVID-19 test results, systemic health conditions, and the presence of pregnancy-related complications both during gestation and at the time of labor and delivery.

Thus, the dental variables included dental care during pregnancy, periodontal status diagnosis (healthy, gingivitis, periodontitis, or periodontal abscess), participation in health promotion and prevention programs, and the type of periodontal therapy received (prophylaxis, scaling, scaling and root planning, or abscess drainage).

All variables were operationally defined and classified according to their nature (qualitative or quantitative) and level of measurement (nominal, ordinal, or ratio).

The data were collected using validated instruments. The dental variables were obtained from the institutional data of EPS Compensar—Oral Health, which were recorded by expert general dentists who had been calibrated based on observation and clinical examination. Maternal and neonatal clinical variables were extracted from institutional medical records, which constitute validated and audited sources. COVID-19 severity was categorized according to the Colombian Infectology Guidelines (2021). Neonatal gestational age was determined using the Modified Ballard Scale. Sociodemographic variables were collected from official records based on the categories established by DANE and the Colombian Ministry of Health.

Regarding blinding, since this was a retrospective study based on the review of medical records, the professionals who provided dental and medical care to the patients did not participate in the research and were unaware of the study objectives; therefore, no observation bias was introduced in the clinical assessments recorded. The investigators responsible for data collection and analysis were trained in the use of the data extraction instrument, applying previously defined standardized criteria. Although complete blinding regarding COVID-19 diagnosis or periodontal status was not possible, objectivity was ensured through cross-verification of records and the exclusive use of documented data from the medical records, without making subjective interpretations.

### 2.4. Analysis of Information

In the initial phase of the study, a descriptive statistics analysis was conducted to provide a comprehensive overview of the distribution and behavior of clinical and obstetric variables. Measures of central tendency and dispersion were calculated for all included variables.

Subsequently, bivariate analyses were performed to explore potential associations between periodontal disease and maternal–neonatal outcomes. The chi-square test was applied to assess statistical significance, and odds ratios (ORs) with 95% confidence intervals were calculated. A *p*-value < 0.05 was considered statistically significant.

In the second phase of the study, which focused on a single clinical setting (HUCSR), due to the greater completeness of the clinical records, certain variables presented zero frequency observations. Given the limited sample size, multivariate statistical analysis was not feasible.

Consequently, advanced mathematical approaches were employed to enrich the analytical framework. Specifically, set theory and probability theory were utilized to explore the relationships among clinical and obstetric variables. Set theory enabled the structuring of data through operations such as union, intersection, complement, and difference [42], facilitating the identification of relevant subsets within the universal set of pregnant women. This logical organization allowed for the construction of quantifiable relationships among dental care, periodontal disease, and COVID-19 diagnosis, in relation to gestational age, neonatal birth weight, and the presence of maternal or neonatal complications.

Additionally, probability theory was applied to estimate the relative frequency of clinical events within the defined sample space. Proportions and conditional probabilities were calculated to evaluate patterns of association among the studied variables [43,44,45]. This mathematical framework integrated both descriptive and probabilistic analysis, thereby enhancing methodological rigor and offering an innovative perspective for interpreting the complex clinical interactions observed in the study.

### 2.5. Defining Sets, Variables, and Operations

For methodological purposes, the study population was initially stratified into two primary groups: pregnant women who did not receive dental care during pregnancy, and those who did. The latter group was further subdivided into four distinct subsets based on specific clinical and dental criteria. In total, sixteen analytical sets were defined and examined in relation to key obstetric variables, including gestational age at birth, neonatal birth weight, and the presence of maternal or neonatal complications during pregnancy or delivery.

The nomenclature and defining characteristics of each set are detailed in Table 1, while the operational definitions and measurement criteria for the analyzed variables are presented in Table 2.

These sets were systematically defined to facilitate the analysis of the application of mathematical operations grounded in set theory and probability.

Using the defined sets, intersections were performed between the *DC* and *DC^C^* sets with respect to the *LBW*, *NBW*, *W*_37_, *W*_38_, *W*_39_, and *W*_40_ sets. Subsequently, considering *DC* as the universal set, representing pregnant women who received dental care, intersections were established between the *CO* and *CO^C^* sets and the same clinical subsets (*LBW*, *NBW*, *W*_37_–*W*_40_). It is important to note that the degree of association derived from set operations was evaluated in relation to the total number of individuals within the universal set defined for each phase of the study.

All analyses were conducted using R software (version 4.5.1) and Microsoft Excel 365.

## 3. Results

### 3.1. Stage 1: Initial Exploratory Descriptive Analysis (5 Clinics)

A total of 156 medical reports of pregnant women who met the inclusion criteria were reviewed. The mean age of the participants was 31.5 years, ranging from 17 to 46 years. The majority of the pregnant women were from Bogotá (*n* = 118), while the remaining 25% (*n* = 33) were from various municipalities in the Andean region of Colombia. The distribution of delivery care was as follows: Los Cobos Medical Center, 30% (*n* = 47); Clínica La Magdalena 27.5% (*n* = 43); Hospital San Rafael, 21.1% (*n* = 33); Hospital San Ignacio, 6.4% (*n* = 10); and in 14.7% of cases (*n* = 23), no information was available.

Regarding COVID-19 test results, 37% (*n* = 58) of the pregnant women tested positive. With respect to vaccination status, only 33.3% (*n* = 52) had received a COVID-19 vaccine, while 66.6% (*n* = 104) had not. The most frequently administered vaccine was Pfizer, received by 21 women (30.8%), followed by Janssen in 14 (20.5%), Moderna in 9 (13.23%), Sinovac in 6 (8.8%), and AstraZeneca in 2 (2.9%).

In relation to systemic history, 14% (*n* = 22) of pregnant women were diagnosed with some form of systemic condition prior to delivery, 3.8% (*n* = 6) had preeclampsia, 3.8% (*n* = 6) hypertension, 1.28% (*n* = 2) hypothyroidism, 1.3% (*n* = 2) obesity, and 0.6% (n = 1) hypotension, 0.6% (*n* = 1) diabetes, 0.6% (*n* = 1) insulin resistance, 0.6% (*n* = 1) Hodgkin lymphoma, 0.6% (*n* = 1) retina left thrombosis and 0.6% (*n* = 1) Sjögren syndrome.

Regarding the socioeconomic status of the pregnant women, the majority belonged to lower strata, distributed as follows: 75% (*n* = 112) in stratum 1, 16% (*n* = 25) in stratum 2, 7.7% (*n* = 12) in stratum 3, and 2 pregnant women with no available information.

With respect to the type of delivery, data were available for 133 pregnant women. Of these, 63.1% (*n* = 84) underwent cesarean section and 36.8% (*n* = 49) had vaginal delivery. In 23.4% (*n* = 19) of the cesarean cases, the reason for the procedure was not recorded, and in 8.6% (*n* = 7), it was performed by personal choice. Among the remaining 58 pregnant women, 10.9% (*n* = 6) experienced complication during vaginal delivery due to fetal distress, 10.9% (*n* = 6) reported failure in labor induction, 10.9% (*n* = 6) had abnormalities in labor dynamics, and 9.09% (*n* = 5) presented edema, proteinuria and hypertensive disorders during pregnancy, delivery, and puerperium. Additionally, 9.09% (*n* = 5) were documented as having received maternal care related to the fetus and the amniotic cavity due to potential delivery complications, recorded in the clinical records as abnormalities in labor dynamics (Table 3).

In reference to the periodontal diagnosis of the pregnant women evaluated, 5.12% (n = 8) were diagnosed with periodontitis, 31.4% (*n* = 49) with gingivitis, and 63.4% (*n* = 99) had no periodontal diagnosis.

#### Bivariate Analysis

When the relationship between periodontal diagnosis and newborn weight was evaluated, it was found that the neonates born to mothers diagnosed with periodontitis had an average birth weight of 2396 kg, while those born to mothers with gingivitis had an average birth weight of 2302 kg. No statistically significant differences were observed between the groups (*p* = 0.45) (Table 4).

On the subject of gestational age, pregnant women diagnosed with periodontitis had an average gestational age of 37.25 weeks, compared to 37.69 weeks in those diagnosed with gingivitis. No significant differences were found between gestational age and periodontal diagnosis (*p* = 0.47) (Table 4).

**Figure 1:**
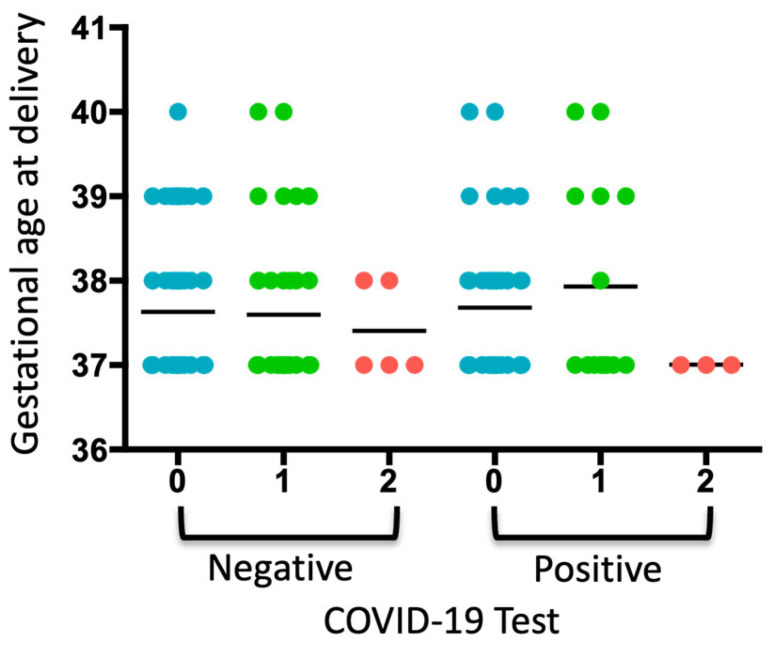
Analysis of the COVID-19 test, gestational age of delivery, and periodontal diagnosis. A dot plot is presented, where each dot represents a pregnant woman included in each group according to whether she tested negative or positive for COVID-19. The numbers indicate the periodontal diagnosis: 0: no periodontal diagnosis, 1: gingivitis, 2: periodontitis. The average of each group is represented.

When analyzing the COVID-19 test results, gestational age at delivery, and periodontal diagnosis, a trend toward increased gestational age was observed in pregnant women diagnosed with gingivitis who tested positive for COVID-19 (Figure 1). Two pregnant women were diagnosed with diabetes, which coincided with a diagnosis of gingivitis. No neonatal or maternal deaths were reported.

Regarding periodontal therapy, *n* = 143 pregnant women (91.6%) did not receive any periodontal treatment, *n* = 10 (6.41%) received prophylaxis, *n* = 2 (1.28%) underwent scaling, and only *n* = 1 (0.64%) received both prophylaxis and scaling. In relation to the prevention and promotion program (P&P), *n* = 36 (23%) did not attend the program, *n* = 78 (50%) did attend, and for *n* = 42 (27%), no information was available.

Concerning the type of periodontal therapy received by pregnant women, no significant differences were found in neonatal weight (*p* = 0.5) or gestational age at delivery (*p* = 0.07) (Table 5).

### 3.2. Stage 2: Analysis Using Set Theory and Probability—Hospital Universitario Clínica San Rafael (HUCSR)

After conducting a comprehensive analysis of medical records in 5 hospitals–clinics in Bogotá, and due to the complexity of the information, it was determined that the HUCSR provided broader and more reliable access to complete clinical data, both from dental care services at Compensar and from the gyneco-obstetric records of the pregnant women themselves. This facilitated the development of the second part of the study.

**Figure 2:**
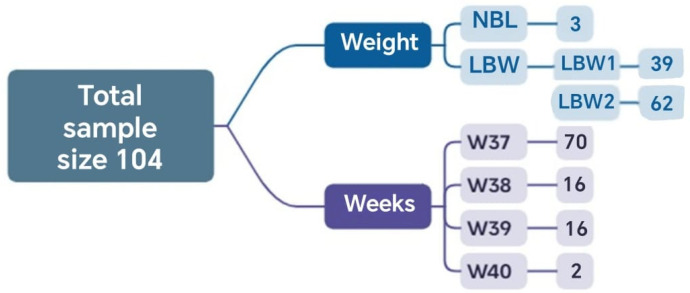
Sets and the corresponding number of elements relative to the total analyzed sample obtained in Phase 1.

In this stage, the results obtained through the application of complementary mathematical tools are presented, highlighting the distribution of cases according to the inclusion criteria, the methodological phases, and the relationship among the defined clinical groups. A total of 104 cases met the established inclusion criteria and had complete information. Of these, 2 reached the 40th week of gestation, and 70 reached the 37th week. Regarding neonatal weight, 3 newborns had normal weight, and 101 had low birth weight (Figure 2).

#### 3.2.1. Phase 1: Analysis and Comparison of Groups of Pregnant Women with and Without Dental Care (DC vs. DC^C^)—HUCSR

When grouping cases according to dental care, 28 belonged to the *DC* set and 76 to the *DC^C^* set. A bivariate analysis based on gestational age revealed a mean of 37.46 weeks with a standard deviation of 0.88 in the *DC* group. In the group of pregnant women without *DC^C^*, the mean was 37.53 weeks with a standard deviation of 0.80. When comparing both groups in terms of gestational age, similar means and medians were observed; however, the group without dental care showed slightly lower variability, suggesting that gestational age in this group was more concentrated around the mean *DC^C^*.

**Figure 3:**
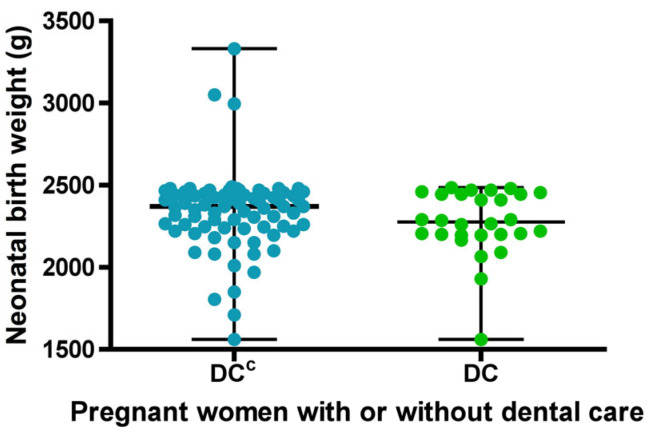
Descriptive statistics of newborn birth weight for sets with (*DC*) or without dental care (*DC^C^*). Each dot represents the newborn birth weight for each study group. The bars indicate the mean and standard deviation.

Regarding the birth weight variable, normality was initially assessed, and according to the Doornik–Hansen test, the distribution was found to be non-normal. In the group of pregnant women who received *DC*, birth weight ranged from 1560 to 2485 g, with a median of 2275 g. In the group without dental care *DC^C^*, birth weight ranged from 1560 to 3330 g, with a median of 2370 g (Figure 3). Although no statistically significant differences were found between the groups in terms of birth weight, there was a tendency for neonates in the group with dental care to have lower weights.

When comparing both groups, pregnant women without dental care showed a higher average birth weight, but a greater dispersion of low-birth-weight data below the mean, compared to those who received dental care, indicating greater variability in the data. (Figure 3).

**Figure 4:**
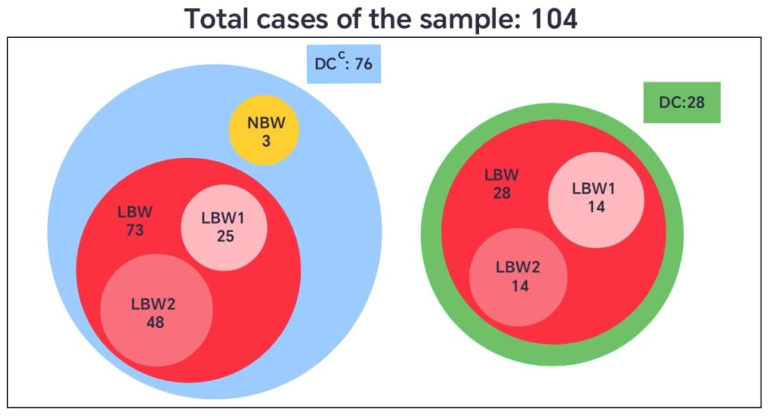
Comparative diagram of neonatal birth weight (*LBW* and *NBW*) among pregnant women with (*DC*) or without dental care (*DC^C^*).

When the birth weight variable was categorized according to the international classification, newborns weighing less than 2500 g were defined as having low birth weight (*LBW*), and those weighing 2500 g or more as normal birth weight (*NBW*). In the group without dental care (*DC^C^*), 73 neonates (96%) presented low birth weight, and 3 (4%) had normal weight. In contrast, all neonates in the group with dental care (*DC*) were born with low birth weight (100%) (Figure 4).

However, considering that the *LBW* category covers a broad range of values (from 1560 g to 2499 g), this set was further divided into two subsets to enhance the understanding of its internal distribution. The subset *LBW*_1_ included newborns weighing 1501 g to 2284 g, and *LBW*_2_ those between 2285 g and 2499 g. This subdivision provided a more detailed representation of the sample, allowing for a finer analysis of clinical patterns within the low-birth-weight group, given that the standard definition encompasses a relatively wide and heterogeneous range (Figure 4).

To identify the conditions under which the neonates were born, considering gestational age, birth weight, and dental care received by the pregnant women, the corresponding intersections between the sets and their complements were analyzed, as shown in Table 6 and Table 7. The intersections revealed that pregnant women without dental care (*DC^C^*) had neonates with higher birth weights compared to those whose mothers received dental care. However, these same women without dental care also showed a greater proportion of low birth weight (*LBW*) neonates than of normal birth weight (*NBW*) neonates, since only three cases of *NBW* were recorded in this group.

The intersections presented in Table 7 show that pregnant women without dental care (*DC^C^*) had a greater proportion of neonates classified as *LBW*_2_ (2285–2499 g), compared with those in the dental care group (*DC*). This indicates that, although the *DC^C^* group included neonates with higher overall birth weights, most of these still fell within the low birth weight range—particularly near the upper limit of 2500 g. In contrast, the *DC* group displayed a more even distribution between the *LBW*_1_ and *LBW*_2_ subsets. These findings suggest that the absence of dental care was associated with a higher concentration of neonates close to the threshold of normal weight.

#### 3.2.2. Phase 2: Analysis of Pregnant Women Who Received Dental Care, in Relation to COVID-19 Status, Gestational Age, Birth Weight, and Presence of Complications. HUCSR

This section presents the findings from the analysis of pregnant women who received dental care (*DC*), with a focus on COVID-19 diagnosis, gestational age, birth weight, and the presence of complications. The distributions of these variables and their potential influence on neonatal outcomes are examined to identify significant patterns.

**Figure 5:**
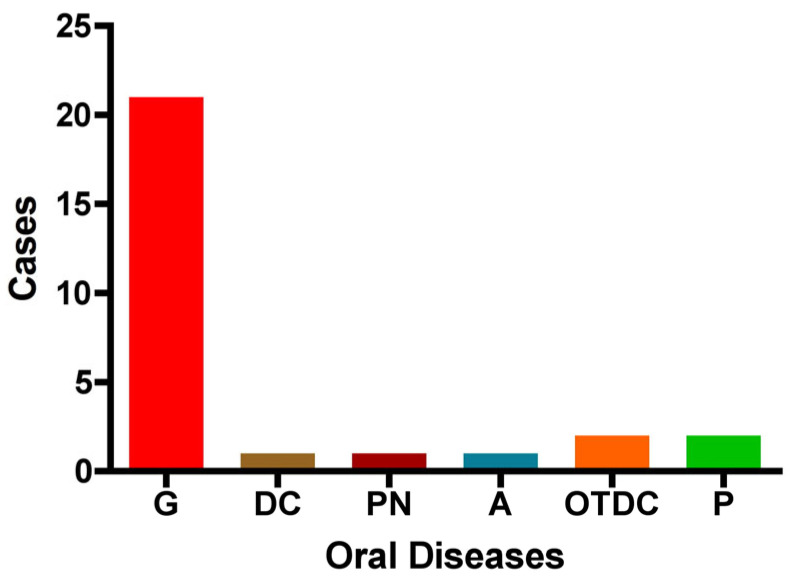
Distribution of oral diseases among the study group. The figure illustrates the number of cases with each condition: G: gingivitis, DC: dentin caries, PN: pulp necrosis, A: absence of dental disease, OTDC: other types of dental caries, and P: pulpitis.

The distribution of oral diseases (*OD*) within the study group is as follows: gingivitis was the most prevalent condition, accounting for 75% of cases (n = 21). Pulpitis and other dental caries were each observed in two cases, while pulp necrosis, dentin caries, and absence of dental pathology were each reported in only one case. These findings indicate that gingivitis is the predominant oral condition among pregnant women in this cohort (Figure 5).

**Figure 6:**
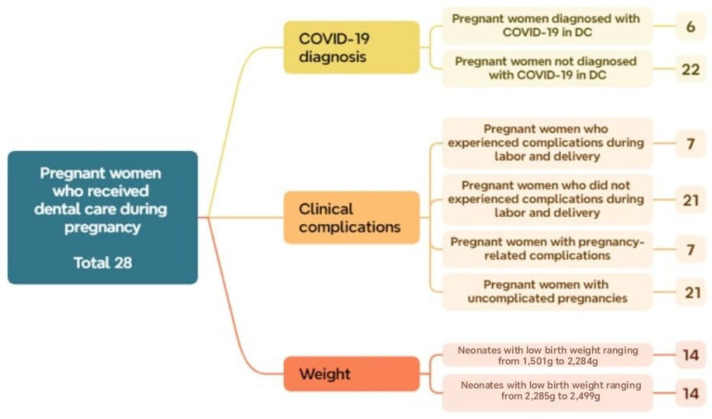
Distribution of subsets and their respective number of cases within the total sample of pregnant women who received dental care during Phase 2.

Additionally, regarding the diagnosis of COVID-19 within this group (*OD*), 78% (22 cases) tested positive, while 22% (6 cases) tested negative. Among the 14 cases that presented some type of *OD*, complications occurred during pregnancy or delivery, indicating that 50% experienced perinatal complications. Regarding birth weight, the sample was evenly distributed across the two low-birth-weight subsets: 14 neonates (50.0%) were classified as *LBW*_1_ and 14 (50.0%) as *LBW*_2_. These findings suggest a higher proportion of pregnant women without a COVID-19 diagnosis and without complications, yet with an equal distribution of neonates within the low-birth-weight category (Figure 6).

**Figure 7:**
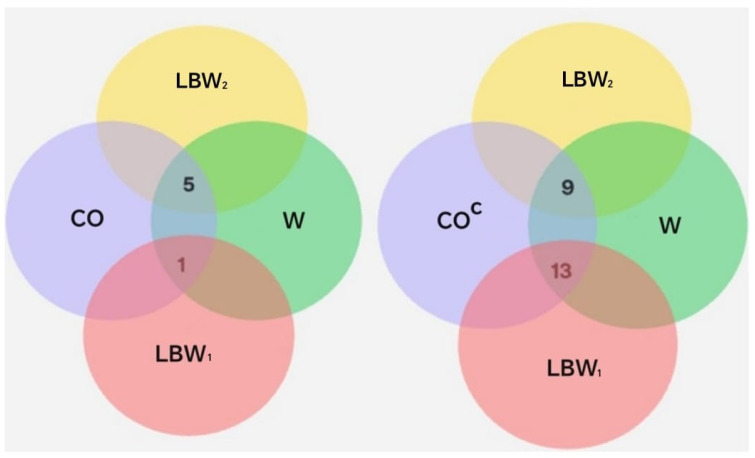
Representation of sets and their intersections. The set *CO* includes individuals diagnosed with COVID-19. The sets *LBW*_1_ and *LBW*_2_ correspond to neonates with low birth weight. *W* denotes the gestational age in weeks at the time of delivery: *W*_37_, *W*_38_, *W*_39_, and *W*_40_.

To explore potential relationships between COVID-19 diagnosis, gestational age, and neonatal birth weight and oral disease, intersections between the relevant sets and their complements were analyzed, as shown in Figure 7 and Table 8.

The results from Phase 2 indicate that, within the analyzed sample, the frequency of neonates classified as *LBW*_2_ (2285–2499 g) was higher among pregnant women diagnosed with COVID-19 compared to those without a positive diagnosis. Additionally, differences were observed in the proportion of COVID-19-positive and -negative cases across the two low-birth-weight subsets. Within the *LBW*_1_ range (1501–2284 g), 13 cases (59.1%) corresponded to neonates born to mothers without a COVID-19 diagnosis, while only 1 case (16.7%) was associated with a COVID-19-positive mother.

In contrast, within the *LBW*_2_ range (2285–2499 g), 9 cases (40.9%) corresponded to neonates born to mothers without a COVID-19 diagnosis, while 5 cases (83.3%) were associated with COVID-19-positive mothers. This indicates a higher proportion of COVID-19-positive cases among neonates in the *LBW*_2_ subgroup, which represents the upper range of low birth weight.

As shown in Table 8, the probabilities were calculated by comparing the number of observed events with the total number of cases, both with and without COVID-19. These results support the finding that pregnant women who tested negative for SARS-CoV-2 and were diagnosed with oral pathologies had a higher proportion of neonates with low birth weight compared to those who tested positive.

**Figure 8:**
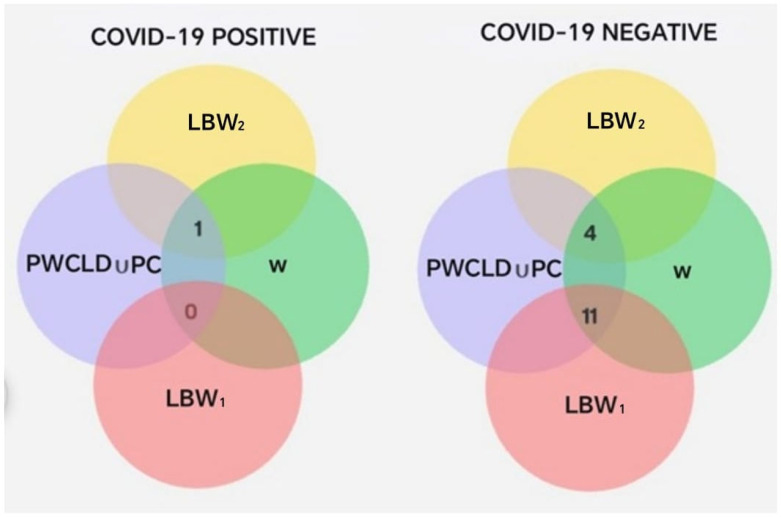
Intersections between PC ∪ PWCLD, W, LBW_1_, and LBW_2_, segmented by COVID-19 diagnosis.

Statistical analysis revealed no significant differences between the groups (*p* = 0.16; chi-square test with Yates correction). To assess the risk of delivering an *LBW*_1_ neonate among COVID-19-positive pregnant women compared to those without a diagnosis, an OR = 0.1385 (95% CI: 0.01376–1.394) was calculated, which was not statistically significant (*p* = 0.1647). On the other hand, the results of the set operations involving COVID-19 diagnosis, pregnancy or delivery complications, oral disease, and low-birth-weight subsets (*LBW*_1_ and *LBW*_2_) are presented (Total = 16) in Table 9 and Figure 8.

The previous intersections show that pregnant women with a negative COVID-19 diagnosis had a higher probability of experiencing complications during pregnancy or delivery (Table 9). In contrast, 83% of pregnant women with a positive COVID-19 diagnosis had an uncomplicated pregnancy and delivery, compared to only 33% among those who tested negative. Additionally, pregnant women with a positive COVID-19 diagnosis showed a higher frequency (Figure 8) of neonates classified within the *LBW*_2_ subset (2285–2499 g), corresponding to the upper range of low birth weight and less delivery-related complications than their COVID-19-negative counterparts.

When evaluating whether there were statistically significant differences between pregnant women diagnosed with COVID-19 and those without, in terms of complications and newborn weight, no significant differences were found (chi-square test with Yates correction, *p* = 0.67). To determine the risk of delivering a low-birth-weight (*LBW*_1_) newborn, among COVID-19-positive pregnant women with complications during pregnancy or delivery, compared to COVID-19-negative women, an OR = 0.1818 was observed (95% CI: 0.0050–6.537), which was not statistically significant (*p* = 0.6250).

## 4. Discussion

The present study employed a quantitative approach with a cross-sectional design and a correlational scope. It was based on the analysis of medical and dental institutional records to explore the potential relationship between periodontal disease and COVID-19 diagnosis in pregnant women, as well as its association with maternal and neonatal outcomes.

In the first phase of this study, no significant differences were observed in the frequency of SARS-CoV-2 infection according to periodontal diagnosis. These findings contrast with those reported by Marouf et al. in 2021 in a case–control study, which identified a significantly higher risk of COVID-19 complications, including assisted ventilation, ICU admission, and even death, among patients with moderate or severe periodontitis compared to those with mild or no periodontitis. This association may be explained by the additional inflammatory burden imposed by oral infection alongside respiratory infection [47,48]. Notably, due to restrictions in dental care during the pandemic, a clinical diagnosis could not be confirmed; therefore, the findings represent an approximation of the phenomenon observed at that time. In contrast, previously published studies were able to conduct detailed clinical evaluations using clinical–periodontal criteria and inflammatory biomarkers [49,50,51].

There is growing evidence that periodontitis may increase the frequency or severity of COVID-19 symptoms. A cross-sectional study reported that patients with both periodontal disease and COVID-19 exhibited salivary levels of IL-6 and experienced a greater number of respiratory symptoms compared to individuals with COVID-19 alone [51]. Additionally, another study confirmed that the presence of periodontitis increases the severity of COVID-19 by approximately 3.7 times and prolongs hospitalization duration, with outcomes influenced by factors such as age, comorbidities, and oral hygiene habits [52]. Moreover, periodontitis shares common risk factors with various chronic inflammatory diseases that have been linked to increased severity of COVID-19. Therefore, it is considered that COVID-19 infection may exacerbate pre-existing conditions and contribute to a worse clinical prognosis. However, in the present study, pregnant women diagnosed with COVID-19 did not exhibit an increased risk of systemic complications attributable to the infection, suggesting that COVID-19 did not directly affect the course of pregnancy. These findings are consistent with those described by Marouf et al., who also emphasize the importance of interpreting such associations with caution and within the appropriate clinical and epidemiological context [47].

Ide and Papapanou in 2013 [53] evaluated the association between periodontal disease and various adverse pregnancy outcomes, including low birth weight (<2500 g), preterm delivery, miscarriage, neonatal death, and preeclampsia. They noted that these conditions may coexist, although it remains unclear whether they share a common etiology [53]. Systematic reviews and meta-analyses have strengthened the evidence supporting this association, consistently demonstrating that periodontal disease is linked to an increased risk of obstetric complications, particularly preterm birth, low birth weight, and preeclampsia [15,54,55,56,57,58,59,60]. However, much of this evidence is derived from observational studies, and the impact of periodontal treatment on the prevention of these adverse outcomes remains unclear. Therefore, incorporating periodontal evaluation into prenatal care is especially relevant, given its potential role in the early identification of modifiable risk factors.

A recent meta-analysis indicated that the severity of periodontitis is associated with an increased risk of adverse COVID-19 outcomes, including higher rates of ICU admissions, severe symptoms, and mortality [49]. Other studies have validated this association through shared inflammatory mechanisms, such as elevated levels of IL-6, C-reactive protein (CRP), D-dimer, and white blood cell count (WBC), as well as common immunological pathways involving NFk-B, NLRP3/IL-1β, and ACE2 expression in periodontal tissues. These mechanisms may contribute to systemic inflammation and exacerbate complications in patients with both conditions [50].

Within the group of oral diseases (ODs), gingivitis was the most prevalent condition, accounting for 75% of cases. The average neonatal weight was similar between pregnant women with gingivitis (2301 g) and those with periodontitis (2396 g). Likewise, the mean gestational age was 37.25 weeks in the periodontitis group and 37.69 weeks in the gingivitis group. These findings differ from those reported by Castaño Suarez et al., who in a meta-analysis identified a moderate association between periodontitis and low birth weight (OR: 2.48) and a weaker association with preterm delivery (OR: 1.87) [61]. However, the meta-analysis showed significant heterogeneity and publication bias, which limit confidence in these results.

Pregnant women with a negative COVID-19 diagnosis were more likely to experience complications during pregnancy or delivery. In contrast, 83% of those who tested positive for COVID-19 had an uncomplicated pregnancy and delivery, compared to only 33% among women who tested negative. Regarding maternal and perinatal outcomes, no significant differences were observed between pregnant women who tested positive for COVID-19 and those who tested negative, in terms of gestational age at delivery, mode of delivery, low birth weight, or the occurrence of neonatal or maternal death. These findings are consistent with those reported in the retrospective comparative study conducted by Cosma et al. in 2022, which found no differences between groups in gestational age at delivery or in the incidence of adverse obstetric outcomes among women with and without SARS-CoV-2 infection [38].

Along these lines, XDyao et al., in a meta-analysis on the impact of the COVID-19 pandemic on preterm birth, reported that, compared to the pre-pandemic period, preterm birth was less frequent during the pandemic. A significant decrease in the likelihood of preterm birth (<37 weeks of gestation; pooled OR = 0.96) and extremely preterm birth (<28 weeks of gestation; pooled OR = 0.92) was observed. The researchers justified this result by relating it to the fact that the COVID-19-related confinement might have led to socio-environmental and behavioral modifications, including a reduction in maternal workload, better air quality, a reduction in non-COVID-19 maternal infections, a reduction in physical activity, and better nutritional support, thus contributing to pregnancy prolongation and exerting a beneficial impact on preterm birth during the pandemic [62].

When analyzing COVID-19 test results, gestational age at delivery, and periodontal diagnosis, a trend toward higher gestational age was observed among pregnant women diagnosed with gingivitis and who tested positive for COVID-19. This observation cannot be directly compared with existing literature, as no studies have simultaneously addressed these three variables. Regarding the association between gingivitis and gestational age alone, the findings of this study are consistent with those reported in a Cuban study that characterized 157 pregnant women with gingival disease. That study found that 40.8% were in the third trimester, with a predominance of mild to moderate gingivitis, and noted that the severity of gingival disease increased with gestational age [63].

This study identified a history of systemic conditions prior to childbirth in the evaluated population, primarily preeclampsia and hypertension. Additionally, two pregnant women were diagnosed with diabetes, both of whom also presented with gingivitis. No neonatal or maternal deaths were reported, which is consistent with findings from the systematic review conducted by Saaqib et al. in 2022 [64]. In that review, an observational study was cited in which five out of eight women with COVID-19 developed proteinuria and hypertension during COVID-19 infection, along with symptoms resembling HELLP syndrome. These manifestations were distinguishable from pregnancy-induced hypertension, as the course of pregnancy normalized following recovery from COVID-19, and signs of preeclampsia and hypertension resolved as the infection subsided [65].

Furthermore, another systematic review reported that pregnancy-related systemic conditions such as gestational diabetes, hypertensive disorders, and preeclampsia were not more frequent among pregnant women diagnosed with COVID-19 compared to those without SARS-CoV-2 infection, which is consistent with the findings of the present study [66]. It has also been noted that COVID-19 symptoms can closely resemble those of preeclampsia, complicating differential diagnosis. In line with previous studies, this research observed that signs of preeclampsia resolved following recovery from SARS-CoV-2 infection, reinforcing the validity of these findings [67].

In the present study, no significant differences were found in the incidence of preterm birth between pregnant women who tested positive for COVID-19 and those in the control group with a negative diagnosis. The variable related to the type of delivery was excluded from the analysis, as in more than 32% of cases, the reason for cesarean section was not specified, whether it was medically indicated or based on the patient’s autonomous decision. The average gestational age of the patients included in the study was 37 weeks. Although no correlation with preterm birth was found, it is clear that these pregnant women did not complete a full-term pregnancy, which is considered to be 39 weeks. While no growth restrictions were diagnosed in the study, it was observed that the newborns had a lower birth weight compared to universal standards for normal and low birth weight. Similar studies have not identified a statistically significant association between COVID-19 infection and most fetal or maternal outcomes, particularly when the infection occurs during early pregnancy (before 20 weeks). However, some reports have documented cases of spontaneous abortion [68].

Despite the findings of the present study, the literature remains heterogeneous. Other reviews, including meta-analysis [28] and retrospective cohort studies [69], have reported increased rates of preterm birth, neonatal ICU admission, and maternal adverse events, including maternal death in women with COVID-19 [69]. These outcomes were particularly observed when infection occurred during the third trimester of pregnancy [28,70], when the disease was moderate to severe [69], or during periods of circulation of more virulent variants such as Delta. Moreover, maternal vaccination has been shown to progressively mitigate these risks in more recent studies [71,72].

Regarding the periodontal therapy received by pregnant women in this study, no significant differences were observed in neonatal birth weight (*p* = 0.50) or gestational age (*p* = 0.07). These findings are consistent with a systematic review conducted in 2017, which concluded that periodontal therapy does not significantly influence the occurrence of preterm birth or low birth weight [39]. In contrast, other randomized clinical trials have reported different outcomes. One study conducted in Chile demonstrated that early periodontal therapy significantly reduced the incidence of preterm birth and low birth weight compared to delayed treatment, in which periodontal care was postponed until after pregnancy [73]. Similarly, another study reported statistically significant differences, with higher birth weight and gestational age observed in the group that received periodontal treatment during pregnancy [74].

The oral conditions identified in this study revealed gingivitis as the predominant disease. Several longitudinal and cross-sectional studies have reported an increased prevalence and severity of gingival disease during pregnancy compared to the postpartum period. Clinically, this increase is manifested by greater gingival inflammation, increased bleeding on probing and brushing, and deeper pre-existing periodontal pockets. The clinical features of biofilm-induced gingival disease and pregnancy-associated gingival disease are largely similar; however, in the latter, patients may develop the condition even with minimal biofilm accumulation, suggesting an exaggerated gingival tissue response to the hormonal changes characteristic of pregnancy [75].

In line with the study’s objective, relevant behavioral patterns were identified across both phases of the analysis. Unlike the exploratory phase, which focused exclusively on data from HUCSR, Phase 1 revealed that although gestational age at birth was similar between groups, but none reached full term and most had newborns with low birth weight; the dental care group exhibited a higher proportion of neonates with low birth weight (100%) compared to the group who did not receive dental care (*DC^C^*) (96%); only 4% had a normal weight. The analysis of the intersections revealed that pregnant women without dental care (*DC^C^*) had neonates with higher birth weights *LBW*_2_ within the low-birth-weight group, compared to those whose mothers received dental care. However, among the low birth weight cases in the *DC^C^* group, there was a greater dispersion of weights below 2000 g. These findings suggest that the absence of dental care was associated with a higher concentration of neonates close to the threshold of normal weight, although the groups were not equivalent, since only 28 reported receiving *DC* compared to 76 who did not. The absence of DC in these patients may have resulted from the lockdown measures implemented during the pandemic.

This observation, which appears to contradict existing literature, may be explained by selection bias. It is possible that pregnant women who received prior dental care had underlying risk factors that warranted closer medical and dental monitoring. Supporting this interpretation, Bobetsis et al. (2020) reported that dental care during pregnancy is more common among women with periodontal disease or other risk factors [40,76].

These results differ from those of earlier clinical trials, which reported that periodontal therapy during pregnancy was associated with a reduction in the incidence of preterm birth and low birth weight [73,74]. However, more recent systematic reviews and meta-analyses have generally concluded that periodontal treatment does not lead to significant differences in neonatal birth weight or gestational age. Moreover, the overall quality of the evidence is considered low to moderate, as most studies are small and their results are heterogeneous [39,77]. In this context, the higher frequency of low birth weight infants in the *DC* group may reflect the influence of uncontrolled confounding factors, such as initial periodontal status, pre-existing maternal conditions (e.g., nutritional status), and sociodemographic characteristics, which were not accounted for in this part of the analysis. These factors, rather than an adverse effect of the treatment itself, may explain the observed findings and are consistent with the heterogeneity reported in the literature.

In Phase 2 of this study, a higher proportion of pregnant women with a positive COVID-19 diagnosis were born with *LBW*_2_ (2285–2499 g), approaching the threshold of normal birth weight. These results support the findings from the exploratory phase of this research and are consistent with reports in the literature [38,68]. However, as previously mentioned, these findings contrast with earlier studies that reported increased risks of hospitalization, ICU admission, and preterm births among pregnant women with COVID-19 [28,78,79].

Conversely, other studies have indicated that in most mild cases, the incidence of severe complications is very low, particularly when pregnant women receive adequate and constant monitoring [80]. The discrepancies observed across studies may be attributed to several factors, including the timing of infection (i.e., the trimester during which COVID-19 was acquired), the presence of maternal comorbidities, access to health care services, vaccination coverage within the studied cohorts [81], and the specific variant circulating at the time of infection [71,72].

Probability represents the likelihood of an event occurring within a specific sample. In this context, an event can be understood as the intersection between sets, which allows us to identify cases in which certain conditions are simultaneously met. This probability was calculated based on the ratio between the total number of possible events and the total number of elements in the sample.

When calculating the probabilities of developing oral pathologies during pregnancy, including gingivitis, along with a negative COVID-19 test, there is a higher likelihood that neonates will be born with low birth weight and a higher probability of experiencing complications during pregnancy or delivery compared to pregnant women who tested positive for COVID-19.

One of the limitations of this study is that the data analyzed correspond to the period between 2020 and 2021, during the initial phase of the COVID-19 pandemic. At that time, there was no comprehensive understanding of the impact of emerging viral variants, nor was there widespread availability or coverage of vaccines, factors that were only achieved in later stages of the pandemic. Consequently, the results presented here may differ from those reported in more recent literature.

Additionally, the sample included a limited number of pregnant women and consisted primarily of patients treated in dental rather than gynecological settings, which may limit the generalizability of the findings to other populations or clinical contexts. All pregnant women who met the inclusion criteria were enrolled without stratification by comorbidities, socioeconomic status, or nutritional habits. This decision was justified by the need to maximize the sample size in a context of limited evidence and restricted access to health and dental services during the COVID-19 pandemic, which may have introduced selection bias and limited the representativeness of the findings.

The retrospective and correlational design employed in this study means that the results should be interpreted as associations rather than causal relationships. Nevertheless, these findings are valuable, as they provide evidence derived from real-world conditions during the health emergency and serve as a foundation for generating new hypotheses that can be tested in multicenter studies with larger sample sizes and through prospective designs or clinical trials. The study was conducted under exceptional conditions, prioritizing the inclusion of all available cases that met the selection criteria to maximize clinically relevant information. Therefore, the results should be interpreted with the understanding that a formal sample size calculation was not feasible at the time due to the absence of prior data in the scientific literature. The analyses were primarily based on univariate and bivariate statistics and mathematical models (sets and probability), without incorporating multivariable models, which limits the ability to adjust for potential confounders and estimate precise effect sizes.

Despite these constraints, the findings provide a useful initial approximation that may guide future research with larger samples and predefined power calculations, more robust designs, longitudinal follow-up, larger samples, and adjusted multivariable analyses to strengthen interpretation and generalizability of the results.

For future research, it is pertinent to explore areas where the evidence remains diverse or contradictory, particularly regarding the influence of the trimester of infection, the clinical severity of COVID-19 disease, the impact of maternal vaccination on perinatal outcomes, and the role of periodontal disease, oral health status, and dental care in the clinical progression of pregnant women.

From a methodological standpoint, this study contributes to an emerging line of research that advocates for the use of mathematical structures to represent and analyze clinical data [82,83]. The findings underscore the value of set theory as a methodological tool for examining phenomena in health-related fields, where identifying relationships among multiple clinical, sociodemographic, and contextual variables in a logical and structured manner is essential. This approach has proven to be an effective alternative for organizing large volumes of data, facilitating comparisons across populations and subpopulations, and enabling analytical models that can be replicated in other contexts of health research, clinical practice, and public health [84,85,86]. Moreover, it offers the potential of transferring findings across regions, pandemic periods, and health conditions, thereby helping to reduce the current fragmentation of evidence and generate more robust conclusions.

Building on this perspective, the theoretical framework proposed in this study aims to explore complex relationships between obstetric and periodontal variables through logical structures that facilitate the organization of clinical information and the identification of visual and intuitive patterns. This model serves as a starting point for generating new hypotheses and guiding future research with experimental, longitudinal, or prospective designs, where early identification of clinical signs could precede adverse outcomes such as low birth weight or preterm delivery [40]. Furthermore, this approach enables simultaneous recording of periodontal data as they occur, allowing for the analysis of exposure to factors such as periodontal disease and COVID-19 infection and their association with pregnancy outcomes. Such representation can be integrated into broader clinical studies, strengthening the understanding of interactions between oral health, COVID-19, and obstetric outcomes.

As demonstrated in this research, mathematical modeling provides a means to cross-reference historical data from significant pandemic events, offering insights that can inform preparedness strategies for future scenarios. Beyond this specific application, the proposed framework can be extrapolated to other clinical domains where multiple biological, behavioral, and environmental variables converge [87]. Its logical structure and capacity to integrate heterogeneous information make it a valuable tool for analyzing complex interactions in fields such as cardiology, endocrinology, or preventive medicine [87], contributing to early risk factor identification and evidence-based intervention strategies. In this regard, the present study positions itself as a pioneering initiative that may open new avenues for interdisciplinary research, aimed at developing predictive models that enhance understanding and prevention.

Beyond its methodological contributions, the proposed mathematical model offers practical advantages for clinicians that extend beyond traditional statistical analyses. First, unlike classical approaches, which often examine variables in isolation, this model integrates clinical, periodontal, gestational, and neonatal factors simultaneously, allowing for dynamic visualization of their interactions. This systemic perspective makes complex relationships easier to interpret and supports early identification of risk combinations without requiring advanced statistical expertise [88].

Second, the model enables real-time incorporation of new data, providing flexibility to update analyses as patient conditions or emerging evidence change, a feature that static statistical methods cannot easily achieve. Third, its ability to simulate hypothetical clinical scenarios, such as variations in periodontal status or infection risk, offers a predictive tool that can inform preventive strategies and individualized care plans.

Finally, by creating a common analytical language, this approach fosters collaboration between dentistry and medicine, strengthening interdisciplinary efforts to improve maternal and neonatal outcomes.

Although no direct relationship was observed between periodontal disease and COVID-19 infection during pregnancy, and dental care, along with other oral conditions, did not appear to significantly influence the control of adverse pregnancy outcomes, future research should focus on optimizing oral health strategies and identifying the most appropriate timing for their implementation across the different stages of pregnancy. This includes addressing various oral infections, such as periodontal disease and respiratory conditions like COVID-19, to improve guidance in the oral healthcare of pregnant women and prevent complications during pregnancy [41], even during the preconception period, which may have a greater clinical impact [40].

This is particularly relevant given that the oral cavity serves as a reservoir for SARS-CoV-2, and periodontal bacteria may facilitate viral infection of oral tissues. Therefore, in addition to antiviral treatment, initial periodontal therapy should be seriously considered and applied periodically [50]. Based on the findings of this study, there is a clear need to establish specific management guidelines to standardize therapeutic strategies for patients presenting with both periodontitis and COVID-19. This would reinforce the integral connection between oral and systemic health, especially in anticipation of future infections with pandemic potential, for which dentists must be adequately prepared.

## 5. Conclusions

These findings suggest that, during pregnancy, periodontal disease and COVID-19 infection may not be directly associated, and that dental care and other oral conditions do not appear to play a significant role in modulating adverse pregnancy outcomes. The average gestational age of the patients included in this study was 37 weeks. Although no association with preterm birth was identified, these pregnancies did not reach full term, which is defined as 39 weeks. Furthermore, while no growth restrictions were diagnosed, the newborns exhibited lower birth weights compared to international standards for normal and low birth weight.

Given that the probabilities observed and reported in this study are derived from hospital medical record data, they should be interpreted with caution due to the limited representativeness of the sample. These estimates may only be extrapolated to other institutions under comparable conditions to those analyzed. From the perspective of the research team, future studies are recommended to increase the sample size and include additional hospitals within the same geographic area.

## Figures and Tables

**Table 1:** Defining sets.

Mathematical Sets	
Name	Description
*DC^C^*	Pregnant women who did not receive dental care during pregnancy. *DC* complement.
*DC*	Pregnant women who received dental care during pregnancy.
*CO*	Pregnant women diagnosed with COVID-19 in *DC*.
*CO^C^*	Complement of *CO*. Pregnant women not diagnosed with COVID-19 in *DC*.
*OD*	Pregnant women diagnosed with oral diseases in DC
*OD^C^*	Pregnant women without any oral disease in *DC*. Complement of *OD*.
*LBW*	Neonates with low birth weight [46].
*LBW*_1_	Neonates with low birth weight ranging from 1501 g to 2284 g.
*LBW*_2_	Neonates with low birth weight ranging from 2285 g to 2499 g.
*NBW*	Neonates with normal birth weight [46].
*W*_37_	Neonates born at 37 weeks of gestation
*W*_38_	Neonates born at 38 weeks of gestation
*W*_39_	Neonates born at 39 weeks of gestation
*W*_40_	Neonates born at 40 weeks of gestation
*PWCLD*	Pregnant women who experienced complications during labor and delivery
*PWCLD^C^*	Pregnant women who did not experience complications during labor and delivery. Complement of *PWCLD*
*PC*	Pregnant women with pregnancy-related complications.
*UCP^C^*	Pregnant women with uncomplicated pregnancies. Complement of *PC*.

**Table 2:** Defining variables.

Variables	
Name	Description
*P*	The variable representing neonatal birth weight was analyzed using the *LBW* and *NBW* sets. Additionally, the LBW set was subdivided into smaller subsets (*LBW*_1_ and *LBW*_2_) to achieve a clearer understanding and more detailed analysis of this group.
*S*	The variable representing gestational age, defined as the number of weeks elapsed until delivery, was analyzed using the *W*_37_, *W*_38_, *W*_39_, and *W*_40_ sets.
*C*	The Complications variable was analyzed using the *PWCLD*, *PWCLD^C^*, *PC*, and *UCP^C^* sets.

**Table 3:** Complications during childbirth (all delivery modes).

Complications of Childbirth	*N* (%)
Fetal distress	6 (10.9%)
Induction failure	6 (10.9%)
Abnormalities in the dynamics of childbirth	6 (10.09%)
Edema, proteinuria, and hypertensive disorders in pregnancy, childbirth, and the postpartum period	5 (9.09%)
Fetal abnormalities and lesions	3 (5.5%)
Fetus and amniotic cavity with possible delivery problems	3 (5.5%)
Unspecified abnormality of the fetus	2 (3.6%)
Infection of the amniotic sac and/or membranes	1 (1.8%)
Complications in labor	1 (1.8%)
Resuscitation in the womb	1 (1.8%)

**Table 4:** Periodontal diagnosis in 156 pregnant women: neonatal weight, gestational age at delivery.

Periodontal Diagnosis (*n* = 156)	Weight of the Newborn (g)	*p* Value
No diagnosis	2302 (SD: 376)	0.45
Gingivitis	2302 (SD: 150)	
Periodontitis	2396 (SD: 98)	
	**Gestational age of delivery** **(weeks)**	
No diagnosis	37.65 (SD: 0.84)	0.47
Gingivitis	37.69 (SD: 1.04)	
Periodontitis	37.25 (SD: 0.46)	

SD: standard deviation.

**Table 5:** Periodontal therapy in 156 pregnant women: neonatal weight, gestational age at delivery.

Periodontal Therapy (156)	Weight of the Newborn (g)	*p* Value
No therapy	2319 (SD: 323)	0.50
Supragingival	2309 (SD: 142)	
SRP	2295 (SD: 106)	
OHI	2180 (SD: 0.00)	
	**Gestational age at delivery** **(weeks)**	
No therapy	37.69 (SD: 0.90)	0.07
Supragingival	37.10 (SD: 0.31)	
SRP	37.00 (SD: 0.0)	
OHI	37.00 (SD: 0.0)	

SD: standard deviation; SRP: root surface instrumentation and scaling. OHI: oral hygiene instruction.

**Table 6:** Neonatal distribution by intersecting conditions: gestational age, birth weight, and dental care with its Probability (*P*%) relative to 104 total cases.

Intersection	${DC}^{c}$(*P*)	*DC*(*P*)	Intersection	${DC}^{c}$(*P*)	DC(*P*)
$W_{37}$ ∩ LBW	49 (0.471)	21 (0.202)	$W_{37}$ ∩ NBW	0 (0.000)	0 (0.000)
$W_{38}$ ∩ LBW	13 (0.125)	2 (0.019)	$W_{38}$ ∩ NBW	1 (0.010)	0 (0.000)
$W_{39}$ ∩ LBW	10 (0.096)	4 (0.038)	$W_{39}$ ∩ NBW	2 (0.019)	0 (0.000)
$W_{40}$ ∩ LBW	1 (0.010)	1 (0.010)	$W_{40}$ ∩ NBW	0 (0.000)	0 (0.000)

“∩” indicates cases that simultaneously meet the three conditions: week of gestation (*W*_37_–*W*_40_) and weight (*LBW*/*NBW*) in the groups of pregnant women with and without dental care.

**Table 7:** Intersections between gestational age subsets (*W*_37_*–W*_40_) and low-birth-weight subsets (*LBW*_1_, *LBW*_2_) in pregnant women with and without dental care (*DC*, *DC^C^*), with their probability (*P*%) relative to 101 total cases.

Intersection	${DC}^{c}$	DC
$W_{37}$ $\;\cap \;{LBW}_{1}$	19 (0.188)	10 (0.099)
$W_{37}$ $\;\cap \;{LBW}_{2}$	30 (0.297)	11 (0.109)
$W_{38}$ $\;\cap \;{LBW}_{1}$	2 (0.020)	1 (0.010)
$W_{38}$ $\;\cap \;{LBW}_{2}$	11 (0.109)	1 (0.010)
$W_{39}$ $\;\cap \;{LBW}_{1}$	4 (0.040)	3 (0.030)
$W_{39}$ $\;\cap \;{LBW}_{2}$	6 (0.059)	1 (0.010)
$W_{40}$ $\;\cap \;{LBW}_{1}$	0 (0.000)	0 (0.000)
$W_{40}$ $\;\cap \;{LBW}_{2}$	1 (0.010)	1 (0.010)

**Table 8:** Distribution of neonates based on the intersection of gestational age (Weeks 37–40) and birth weight (*LBW*/*NBW*) within the COVID-19 group (*CO*) and its complement (*CO^C^*), with its probability (*P*) relative to 28 total cases.

Intersection	*CO*	*CO^C^*	Intersection	*CO*	*CO^C^*
$W_{37}$ $\;\cap \;{LBW}_{1}$	1 (0.036)	0 (0.000)	$W_{37}$ $\;\cap \;{LBW}_{2}$	5 (0.178)	0 (0.000)
$W_{37}$ $\;\cap \;{LBW}_{1}$	0 (0.000)	9 (0.321)	$W_{37}$ $\;\cap \;{LBW}_{2}$	0 (0.000)	6 (0.214)
$W_{38}$ $\;\cap \;{LBW}_{1}$	0 (0.000)	1 (0.036)	$W_{38}$ $\;\cap \;{LBW}_{2}$	0 (0.000)	1 (0.036)
$W_{39}$ $\;\cap \;{LBW}_{1}$	0 (0.000)	3 (0.107)	$W_{39}$ $\;\cap \;{LBW}_{2}$	0 (0.000)	1 (0.036)
$W_{40}$ $\;\cap \;{LBW}_{1}$	0 (0.000)	0 (0.000)	$W_{40}$ $\;\cap \;{LBW}_{2}$	0 (0.000)	1 (0.036)

“∩” Indicates cases that simultaneously meet all three conditions. The first number corresponds to cases, and the number in parentheses is the probability. This probability was calculated based on the ratio between the total number of possible events and the total number of elements in the sample.

**Table 9:** Set intersection involving COVID-19 diagnosis, perinatal complications, neonatal birth weight, and gestational age at delivery.

Intersection	*LBW*_1_	*LBW*_2_
${CO}^{c}$ ∩ *PC* ∩ $W_{37}$	5 (0.013)	1 (0.063)
${CO}^{c}$ ∩ *PWCLD* ∩ $W_{37}$	3 (0.188)	1 (0.063)
${CO}^{c}$ ∩ *PWCLD* ∩ $W_{38}$	0 (0.000)	1 (0.063)
${CO}^{c}$ ∩ *PWCLD* ∩ $W_{39}$	3 (0.188)	1 (0.063)
CO ∩ *PC* ∩ $W_{37}$	0 (0.000)	1 (0.063)

“∩” Indicates cases that simultaneously meet all three conditions. The first number corresponds to cases, and the number in parentheses is the probability. This probability was calculated based on the ratio between the total number of possible events and the total number of elements in the sample.

## Data Availability

Some data are stored inth repository of the Central Library at Pontificia Universidad Javeriana. The Excel files obtained from medical records are kept on computers at Pontificia Universidad Javeriana, and the datasets generated and reported here are also stored on the University´s computers.
